# Validation of the Trifecta Scoring Metric in Vacuum-Assisted Mini-Percutaneous Nephrolithotomy: A Single-Center Experience

**DOI:** 10.3390/jcm11226788

**Published:** 2022-11-16

**Authors:** Efrem Pozzi, Matteo Malfatto, Matteo Turetti, Carlo Silvani, Letizia Maria Ippolita Jannello, Susanna Garbagnati, Gilda Galbiati, Stefano Paolo Zanetti, Fabrizio Longo, Elisa De Lorenzis, Giancarlo Albo, Andrea Salonia, Emanuele Montanari, Luca Boeri

**Affiliations:** 1 Department of Urology, Foundation IRCCS Ca’ Granda—Ospedale Maggiore Policlinico, 20122 Milan, Italy; 2 Department of Clinical Sciences and Community Health, University of Milan, 20122 Milan, Italy; 3 Division of Experimental Oncology/Unit of Urology, URI, IRCCS Ospedale San Raffaele, University Vita-Salute San Raffaele, 20132 Milan, Italy

**Keywords:** percutaneous nephrolithotomy, vacuum-assisted percutaneous nephrolithotomy, stone-free rate, complications, trifecta

## Abstract

Background: Scoring metrics to assess and compare outcomes of percutaneous nephrolithotomy (PCNL) are needed. We aim to evaluate prevalence and predictors of trifecta in a cohort of patients treated with vacuum-assisted mini-percutaneous nephrolithotomy (vmPCNL) for kidney stones. Methods: Data from 287 participants who underwent vmPCNL were analysed. Patients’ and stones’ characteristics as well as operative data were collected. Stone-free was defined as no residual stones. The modified Clavien classification was used to score postoperative complications. Trifecta was defined as stone-free status without complications after a single session and no auxiliary procedures. Descriptive statistics and logistic regression models tested the association between predictors and trifecta outcome. Results: After vmPCNL, 219 (76.3%) patients were stone-free, and 81 (28.2%) had postoperative complications (any Clavien). Of 287, 170 (59.2%) patients achieved trifecta criteria. Patients who achieved trifecta status had smaller stone volume (*p* < 0.001), a higher rate of single stones (*p* < 0.001), shorter operative time (*p* < 0.01), and a higher rate of single percutaneous tract (*p* < 0.01) than −trifecta patients. Trifecta status decreased with the number of calyces involved, being 77.1%, 18.8%, and 4.1% in patients with 1, 2, or 3 calyces with stones, respectively (*p* < 0.001). Multivariable logistic regression analysis showed that stone volume (OR 1.1, *p* = 0.02) and multiple calyces being involved (OR 2.8 and OR 4.3 for two- and three-calyceal groups, respectively, all *p* < 0.01) were independent unfavourable risk factors for trifecta after accounting for age, BMI, gender, operative time, and number of access tracts. Conclusions: Trifecta status was achieved in 6 out of 10 patients after vmPCNL. Stone distribution in multiple calyceal groups and stone volume were independent unfavourable risk factors for trifecta.

## 1. Introduction

Percutaneous nephrolithotomy (PCNL) is an endourological technique globally accepted as the treatment of choice for kidney stones larger than 20 mm in adults [1,2]. PCNL was found to be effective in stone treatment [3], but its use has been limited by possible serious postoperative complications, including fever (10.8%), bleeding requiring transfusion (7%), organ injury (0.4%), thoracic complications (1.5%), and sepsis (0.5%) [4].

In recent years, technological developments have led to the miniaturisation of instruments (“mini-perc”, “ultra-mini perc”, and “micro-perc”) to reduce PCNL-related morbidity, but this comes with several drawbacks in terms of procedural outcomes. In fact, miniPCNL may be limited by difficulty in stone removal, reduced visibility, longer operative time and higher intraoperative renal pressures [5]. Recently, aspiration-assisted devices have been introduced in PCNL to overcome the limitations of miniaturised instruments while achieving comparable results in terms of safety and efficacy [6].

The vacuum-assisted access sheath (ClearPetra; Well Lead Medical, Guangzhou, China) is among the latest introductions into the PCNL armamentarium [7,8]. Previous studies have shown that vacuum-assisted miniPCNL (vmPCNL) was associated with higher stone-free rates, lower rates of infectious complications, shorter operative time, and reduced hospitalisation costs than classic miniPCNL [8,9]. However, it is difficult to effectively compare outcomes between different procedures if precise scoring metrics are lacking.

In fact, with the increasing number of techniques and equipment that can be referred to as PCNL or miniPCNL which are proposed to improve treatment efficacy, there is a clinical need for a validated tool to standardise and compare outcomes in terms of stone-free rate and complications.

EL-Nahas et al. proposed the “trifecta” criteria to evaluate outcomes in miniPCNL. Trifecta was defined as stone-free status without complications after a single session of surgery [10]. Authors investigated trifecta in a cohort of miniPCNL, but this scoring metric has never been explored in vmPCNL.

Therefore, aims of this study were: (i) to validate the trifecta criteria in vmPCNL and (ii) to investigate potential predictors of trifecta in a cohort of patients treated with vmPCNL for kidney stones.

## 2. Materials and Methods

Data from 315 patients who underwent vmPCNL in our tertiary-referral academic centre between June 2016 and September 2021 were retrospectively analysed.

Exclusion criteria were: congenital renal or skeletal anomalies (*n* = 17); procedures with large stone volume and planned staged procedures (*n* = 30); endoscopic combined intrarenal surgery procedures (*n* = 2).

Patients’ characteristics were collected, and the Charlson comorbidity index (CCI) was used to score health comorbidities [11]. The CCI was categorised as 0 vs. ≥1. Each patient underwent a preoperative urographic computed tomography (CT) scan, which was used to collect stone parameters, such as stone volume, location, side, burden (single, multiple, or staghorn), estimation of density (Hounsfield unit-HU [12,13]), and the number of calyceal groups affected by stones. The ellipsoid formula (length × width × height × π × 1/6) was used to calculate stone volume [14]. The number of affected calyces was one when one major calyceal group or the renal pelvis were affected, two for two groups, and three when all the major calyceal groups (upper, middle, and lower) were affected. Stone characteristics were evaluated with CT images before surgery by the treating urologists. Two experienced (>150 PCNL performed) endourologists (E.M.; F.L.) performed vmPCNL in a standardised fashion.

### 2.1. Surgical Technique

vmPCNL were performed with general anaesthesia and the patient in the supine Valdivia position. The 16 Ch ClearPetra set (namely, vmPCNL), the 12 Ch MIP nephroscope, and the holmium laser (VersaPulse PowerSuite 100 W, Lumenis, Yokne’am Illit, Israel) were used during surgery. Renal puncture was performed with combined fluoroscopic and ultrasonographic control. One-shot tract dilation [15] was performed with the CleaPetra set. For irrigation, a saline gravity bag located about 1.5 m above patient level was used. After stone fragmentation, fragments were removed through the aspiration-assisted sheath. An 8 Ch nephrostomy tube was placed as an exit strategy in all cases; conversely, the ureteral catheter, used for retrograde pyelography before kidney access, was left in place or removed according to the surgeon’s decision.

### 2.2. Postoperative Evaluation

The number of the percutaneous tracts and operative time (OT) were recorded. Postoperative management included: the bladder catheter was removed after 24 h, and the nephrostomy tube was closed the same day; after 48 h, an antegrade pyelography was performed to assess ureteral canalisation and the presence of residual stones. The nephrostomy tube was removed in case of normal pyelography. On postoperative day three patients were discharged.

The PCNL-adjusted Clavien Score was used to score complications [16]. For the specific purpose of this study, for every patient we recorded each complication with its severity, and the highest Clavien Score was reported [17].

A CT scan was requested within 3 months after vmPCNL to look for residual stones. The stone free rate (SFR) was considered as the absence of residual fragments [18]. According to the volume of residual stones, observation or invasive procedures (mPCNL, extracorporeal shockwave lithotripsy, or retrograde intrarenal surgery) were proposed.

As previously described, trifecta was defined as stone-free outcome without complications after a single session and no auxiliary procedures [10].

Data collection adhere to the principles of the Declaration of Helsinki. All patients signed an informed consent agreeing to share their own anonymous information for future studies. The study was approved by the Foundation IRCCS Ca’ Granda—Ospedale Maggiore Policlinico Ethical Committee (Prot. 25508).

### 2.3. Statistical Analysis

The Shapiro–Wilk test was used to test normality of data. Data are presented as medians (interquartile range; IQR) or frequencies (proportions). Clinical parameters and intraoperative and postoperative characteristics were compared between patients who achieved trifecta (+trifecta) and those who did not (−trifecta) with the Mann-Whitney test and Fisher exact test, as indicated.

Univariable and multivariable logistic regression models were used to investigate potential predictors of −trifecta status. Statistical analyses were performed using SPSS v.26 (IBM Corp., Armonk, NY, USA). All tests were two-sided, and statistical significance level was determined at *p* < 0.05.

## 3. Results

After exclusion criteria, 287 patients were considered and included in the study.

Table 1 reports clinical parameters and perioperative variables. Overall, median (IQR) age and BMI were 56 (47–65) years and 24.6 (22.0–27.7) kg/m^2^, respectively. Multiple stones were found in 191 (66.6%) patients, and the median stone volume was 2.2 (1.0–4.6) cm^3^. Stones were located in 2 and ≥3 calyces in 79 (27.5%) and 26 (9.1%) patients, respectively. Median operative time was 107 (80–140) min, and multiple access tracts were performed in 49 (17.1%) cases. In total, 81 (28.2%) patients had postoperative complications (any Clavien Dindo). A detailed characterisation of post vmPCNL complication was reported in Supplementary Table S1.

Trifecta status was achieved in 170 (59.2%) cases. Among patients who did not achieve trifecta, 83 (70.9%) had postoperative complications, 51 (4.3%) required a second look or auxiliary procedures, and 51 (43.6%) were not stone-free. Median size of residual fragments was 5 (4–10) mm. In total, 62 (53.0%), 40 (34.2%) and 15 (12.8%) participants had 1, 2, and 3 criteria, respectively, for being considered as −trifecta.

Patients who achieved trifecta status had smaller stone volume (1.9 (0.9–3.1) cm^3^ vs. 2.9 (1.2–7.8) cm^3^, *p* < 0.001), a higher rate of single stones (44.1% vs. 17.9%, *p* < 0.01) and a higher rate of single percutaneous tract (91.2% vs. 70.9%, *p* < 0.001) than −trifecta participants (Table 2). Operative time was shorter in +trifecta patients (90 vs. 120 min., *p* < 0.001) than −trifecta (Table 2). Receiver operating characteristic (ROC) curve revealed that a stone volume cutoff value of 1.5 cm^3^ could predict trifecta achievement with 76.3% sensitivity and 74.1% specificity.

Trifecta status decreased with the number of calyces involved, being 77.1%, 18.8% and 4.1% in patients with 1, 2, or ≥3 calyces with stones, respectively (*p* < 0.001). Length of stay was shorter for trifecta group (4 vs. 6 days, *p* < 0.001). Groups were similar in terms of age, BMI, CCI, and surgeon experience (Table 2).

Univariable logistic regression analysis showed that female gender (OR 1.8, *p* = 0.01), higher stone volume (OR 1.2, *p* < 0.001), operative time (OR 1.0, *p* < 0.01), procedure with multiple access tracts (OR 4.5, *p* < 0.001), and multiple calyces involved (OR 3.8 for 2 calyces and 6.9 for ≥3 calyces, (all *p* < 0.001) were all associated with −trifecta status (Table 3). Multivariable logistic regression analysis revealed that stone volume (OR 1.1, *p* = 0.02) and multiple calyces involved (OR 2.8 and OR 4.3 for two- and three-calyceal groups, respectively, all *p* < 0.01) were independent risk factors for −trifecta after accounting for age, BMI, gender, operative time, and number of access tracts.

## 4. Discussion

This study was specifically designed to investigate the effectiveness of vmPCNL for the treatment of kidney stones by means of trifecta status, i.e., SFR, no complications after a single session, and no auxiliary procedures. We found that trifecta was achieved in six out of ten patients in our cohort and that higher stone volume, along with multiple calyces involved, were negative predictors of trifecta status.

MiniPCNL is currently recognised as one of the standard treatment options in the field of stone surgery [19]. In recent years, technological developments have been introduced to increase performance and reduce the burden of the procedure. However, limited scoring metrics are used in clinical practice to objectively evaluate miniPCNL outcomes in terms of SF status and complications. Recently, EL-Nahas et al. proposed the trifecta scoring as a method of standardising miniPCNL outcomes [10]. To the best of our knowledge, the trifecta metric has never been validated in vmPCNL series, which is one of the latest technological evolutions in percutaneous stone surgery.

Previous studies have shown the efficacy and safety of vmPCNL for renal stone treatment. Lai et al. analysed and compared a series of 75 participants who underwent vmPCNL to 75 patients treated with PCNL with a peel-away access sheath. Authors found higher stone-free rates but shorter operative times and lower rates of infection after vmPCNL [7]. Lievore et al. found lower rates of infectious complications, shorter OT, and reduced radiation exposure in 104 patients treated with vmPCNL compared to 52 patients who underwent miniPCNL [8]. Recently, a meta-analysis conducted by Zhu et al. demonstrated an improvement in safety and efficiency in procedures with a vacuum-assisted sheath compared to those with a conventional sheath [20]. These results highlighted higher SFR while reducing operative time and postoperative infection by using vacuum-assisted technology.

In light of the emerging evidence supporting a clinical benefit of vmPCNL compared to classic miniPCNL, this procedure has never been validated in terms of objective metrics, such as trifecta status.

EL-Nahas et al. were the first to introduce trifecta scoring in PCNL [10]. Authors analysed 944 patients submitted to miniPCNL and found that trifecta was achieved in 84% of cases. Independent unfavourable risk factors were number of calyceal groups affected by the stones and number of percutaneous tracts [10]. In the current series, trifecta status was achieved in approximately 60% of cases. Our results show that patients who achieved trifecta status had smaller stone volume, a higher rate of single stones, and fewer calyces involved than −trifecta participants, suggesting that the more complex the stone, the more difficult is to obtain trifecta in vmPCNL. This finding is also supported by previous literature in which calyceal stone distribution was found to be a significant predictor of SFR after PCNL [21]. Similarly, the number of calyces involved emerged as a risk for complications in standard PCNL [22]. This can be explained by some fragments in calyces away from the percutaneous access being missing or by increasing the risks of complications with multiple punctures. In fact, endoscopic combined intrarenal surgery (ECIRS) has been found to be a safe and effective procedure for treating large and complex renal stones [23]. A systematic review and meta-analysis showed that ECIRS had higher one-step SFR and lower complications compared to PCNL for complex stones [23]. Of clinical note, the identification of residual fragments that cannot be achieved with the nephroscope is achieved through the combination of both retrograde and antegrade approaches, thus improving SFR. Moreover, the combined approach reduces the need for multiple kidney access, with consequent lower rates of complications. As a whole, ECIRS could be the technique of choice in case of large stones or stones in multiple calyces.

We also found that operative time and length of stay were shorter in +trifecta patients than in those who did not achieve trifecta status. Therefore, trifecta achievement gains even more importance in terms of clinical outcomes and reduction of hospitalisation costs. It should be mentioned that the hospitalisation time for PCNL in our study was longer compared to other reports in which length of stay for uncomplicated PCNL is progressively shortening [24]. Several procedural and management-related factors were associated with longer hospitalisation time: failed ureteral canalisation during antegrade pyelography on day 2 was usually managed with repeated pyelography the next day; bleeding from the nephrostomy tube or urethra was managed with observation and laboratory testing; fever was managed with parental antibiotics in accordance with the Infections Disease department (in few cases with treatment >10 days). As a whole, patients with longer hospitalisation were those with higher severity of complications; two patients had postoperative sepsis and were treated with 3 weeks of parenteral antibiotics, two patients had urine leakage and were treated with retrograde stenting, and three patients had postoperative bleeding and underwent embolisation.

This study provides a standardised definition for the global outcome of kidney stone treatment (among which the most important are SFR and complication), that can be applicable to any procedure (standard, mini, micro PCNL). From a clinical and scientific point of view, standardisation of outcome evaluation for any surgical intervention is important for comparing different procedures and tailoring the best treatment for each patient. For instance, the identification of clinical characteristics not associated with trifecta achievement in miniPCNL could change the treatment plan to different procedures (e.g., standard PCNL with ballistic energy) or the postoperative care in order to reduce potential PCNL-related complications (e.g., extended antibiotic prophylaxis, stone culture, DJ positioning). Alternatively, −trifecta patients could be identified as those who might benefit from more intense follow-up imaging (CT-based) or immediate second-look surgery to achieve stone-free status.

As compared to published data concerning SFR after PCNL (range 86–94%), this study showed a lower rate of stone-free status (76.4%), which is similar to that of retrograde intrarenal surgery (RIRS) (61–80%) [25,26,27]. Of note, it was consistently reported that RIRS was associated with shorter hospitalisation time, lower rates of complications, and acceptable efficacy than PCNL. Therefore, it should be considered as an alternative treatment option in this group of patients [26]. However, in the previous series, cases with residual fragments of <4 mm were considered stone-free, thus partially explaining the difference in SFR with our series.

This study is innovative because it is the first in the published literature to investigate and validate trifecta scoring in vmPCNL, which is the most innovative armamentarium of miniaturised PCNL. The second strength of the study is that we have analysed a homogenous cohort of patients with a thorough clinical and perioperative evaluation. In particular, SFR in our study was based on a CT scan performed within 3 months after the procedure; conversely, EL-Nahas et al. used plain X-ray in 85% of cases [10]. This could be the reason for the higher SF (90% vs. 76%) and trifecta rates (86% vs. 60%) observed in their cohorts compared to our study.

Another limitation of this study is the single-centre-based and retrospective nature of the study’s design, which raises the possibility of selection biases. Therefore, future studies should externally validate our findings.

## 5. Conclusions

This study reveals that trifecta (namely stone free, no complications, in a single session without additional procedures) can be achieved in approximately six out of ten patients after vmPCNL. Stones distribution in multiple calyceal groups and stone volume are independent unfavourable risk factors for trifecta. In the future, larger prospective studies are needed to validate our findings.

## Figures and Tables

**Table 1:** Demographic characteristics of the whole cohort (*n* = 287).

Age (Years)	
Median (IQR)	56.0 (47–65)
Range	19–84
Male Gender (No. (%))	175 (61.0)
BMI (kg/m^2^)	
Median (IQR)	24.6 (22.0–27.7)
Range	17.9–46.1
CCI (score)	
Median (IQR)	0.0 (0.0)
Mean (SD)	0.6 (0.2)
Range	0–6
CCI ≥ 1 (No. (%))	105 (36.6)
Laterality (No. (%))	
Right	137 (47.7)
Left	150 (52.3)
Stone volume (cm^3^)	
Median (IQR)	2.2 (1.0–4.6)
Range	0.5–26.3
Single stone [No. (%)]	96 (33.4)
Stone density (Hounsfield unit)	
Median (IQR)	1280 (880–1423)
Range	100–2286
Number of affected calyces (No. (%))	
Single or pelvis	182 (63.4)
2 calyces	79 (27.5)
≥3 calyces	26 (9.1)
Multiple access tracts (No. (%))	49 (17.1)
Operative time (min)	
Median (IQR)	107 (80–140)
Range	36–255
Hospitalisation time (days)	
Median (IQR)	4.0 (3.0–6.0)
Range	2.0–22.0
Postoperative complications (No. (%))	
(Highest Clavien score)	
Clavien–Dindo I	22 (7.7)
Clavien–Dindo II	45 (15.7)
Clavien–Dindo IIIa/b	14 (4.9)
Stone free rate (No. (%))	219 (76.3)
Trifecta achieved (No. (%))	170 (59.2)

Keys: BMI = body mass index; CCI = Charlson Comorbidity Index.

**Table 2:** Descriptive statistics of the whole cohort as segregated according to trifecta achievement (*n* = 287).

	+Trifecta	−Trifecta	*p*-Value *
Number of patients (No. (%))	170 (59.2)	117 (40.8)	
Age (years)			0.1
Median (IQR)	56.0 (47–67)	54.0 (46–63)	
Range	19–84	19–83	
Male Gender (No. (%))	94 (53.7)	81 (46.3)	0.02
BMI (kg/m^2^)			0.5
Median (IQR)	24.6 (21.8–28.0)	24.6 (22.0–27.2)	
Range	17.9–46.1	18.9–42.2	
CCI (score)			0.4
Median (IQR)	0.0 (0.0)	0.0 (0.0)	
Mean (SD)	0.4 (0.2)	0.5 (0.2)	
Range	0–4	0–6	
CCI ≥ 1 (No. (%))	59 (34.7)	46 (39.3)	0.2
Laterality (No. (%))			0.1
Right	88 (51.8)	49 (41.8)	
Left	82 (48.2)	68 (58.1)	
Stone volume (cm^3^)			<0.001
Median (IQR)	1.9 (0.9–3.1)	2.9 (1.2–7.8)	
Range	0.5–21.2	0.5–26.3	
Single stone (No. (%))	75 (44.1)	21 (17.9)	<0.01
Stone density (Hounsfield unit)			0.1
Median (IQR)	1241 (850–1400)	1300 (960–1500)	
Range	100–2286	400–2230	
Number of affected calyces (No. (%))			<0.001
Single or pelvis	131 (77.1)	51 (43.5)	
2 calyces	32 (18.8)	47 (40.2)	
≥3 calyces	7 (4.1)	19 (16.2)	
Multiple access tracts (No. (%))	15 (8.8)	34 (29.1)	<0.001
Operative time (min)			<0.001
Median (IQR)	90.0 (70–125)	120 (80–155)	
Range	36–245	40–255	
Hospitalisation time (days)			<0.01
Median (IQR)	4.0 (3.0–5.0)	6.0 (4.0–9.0)	
Range	2.0–21.0	2.0–22.0	

Keys: BMI = body mass index; CCI = Charlson Comorbidity Index; * *p* value according to the Mann–Whitney test and Fisher Exact test, as indicated.

**Table 3:** Logistic regression models predicting unfavourable trifecta achievement (−trifecta).

	UVA Model	MVA Model
	**OR;** *p* **-Value [95% CI]**	**OR;** *p* **-Value [95% CI]**
Age	0.98; 0.11 [0.97–1.01]	0.98; 0.36 [0.95–1.02]
BMI	0.97; 0.27 [0.92–1.02]	0.94; 0.17 [0.87–1.03]
CCI ≥ 1	1.31; 0.21 [0.81–2.14]	
Female Gender	1.81; 0.01 [1.10–2.98]	1.76; 0.11 [0.87–3.68]
(vs. Male)		
Stone Volume	1.17; <0.001 [1.07–1.27]	1.12; 0.02 [1.02–1.24]
Stone density (HU)	1.01; 0.11 [0.98–1.06]	
*n*. of involved calyces		
Single/Renal pelvis	Ref.	Ref.
2 calyces	3.80; <0.01 [2.14–6.78]	2.84; 0.01 [1.19–6.77]
≥3 calyces	6.93; <0.001 [2.58–9.56]	4.31; 0.01 [1.19–9.32]
Multiple access tracts	4.47; <0.001 [2.29–8.74]	1.54; 0.47 [0.47–5.06]
Operative time	1.01; <0.01 [1.01–1.05]	1.01; 0.28 [0.99–1.01]

Keys: UVA = univariate model; MVA = multivariate model, BMI = body mass index; CCI = Charlson Comorbidity Index; HU = Hounsfield Unit.

## Data Availability

Data available on request due to restrictions.

## Supplementary Materials

The following supporting information can be downloaded at: https://www.mdpi.com/article/10.3390/jcm11226788/s1, Table S1: Detailed characterisation of postoperative complications in the whole cohort (*n*, %).
