# Supplementary material for: Validation of the Trifecta Scoring Metric in Vacuum-Assisted Mini-Percutaneous Nephrolithotomy: A Single-Center Experience
Source: J Clin Med. 2022 Nov 16;11(22):6788. doi: 10.3390/jcm11226788 (PMC9697932; doi:10.3390/jcm11226788)
Supplement: Supplementary file 1 [file jcm-11-06788-s001.zip › jcm-2017211-supplementary.pdf]

Journal of
Clinical Medicine

MDPI

Supplementary Materials

Table S1. Detailed characterization of postoperative complications in the whole cohort (n, %).

Complication type	Clavien-Dindo I	Clavien-Dindo II	Clavien-Dindo IIIa	Clavien-Dindo IIIb
Pain	15 (5.2)			
Bleeding	6 (2.1)	10 (3.5)	3 (1.0)	3 (1.0)
Infectious	5 (1.7)	39 (13.6)	1 (0.3)	
Leakage	3 (1.0)			
Drainage	5 (1.7)		5 (1.7)	
Renal failure	1 (0.3)			
Other	1 (0.3)	1 (0.3)	1 (0.3)	1 (0.3)
